# Cholesterol Metabolism in Pancreatic Cancer

**DOI:** 10.3390/cancers15215177

**Published:** 2023-10-27

**Authors:** Artur Rebelo, Jörg Kleeff, Yoshiaki Sunami

**Affiliations:** Department of Visceral, Vascular and Endocrine Surgery, University Medical Center Halle, Martin-Luther-University Halle-Wittenberg, 06120 Halle, Germany; artur.rebelo@uk-halle.de (A.R.); joerg.kleeff@uk-halle.de (J.K.)

**Keywords:** pancreatic cancer, lipid metabolism, cholesterol metabolism, mevalonate pathway, lipoprotein, clinical trial

## Abstract

**Simple Summary:**

This review delves into metabolic reprogramming in pancreatic cancer and its development, with a special emphasis on the mevalonate pathway, encompassing cholesterol biosynthesis, transport, targeting approaches, and clinical investigations. It describes how cancer cells manipulate cholesterol metabolism to fuel their proliferation, outlining the specific metabolic routes employed by pancreatic cancer cells for cholesterol production and the potential of inhibiting these processes to decelerate cancer progression. The paper elucidates intracellular cholesterol storage, inter-cellular transport, and its correlation with cancer metastasis. Moreover, the review highlights promising pharmaceutical candidates for pancreatic cancer therapy. Overall, this comprehensive review provides valuable insights into the prospects of combating pancreatic cancer by selectively addressing cholesterol-related processes.

**Abstract:**

Pancreatic cancer’s substantial impact on cancer-related mortality, responsible for 8% of cancer deaths and ranking fourth in the US, persists despite advancements, with a five-year relative survival rate of only 11%. Forecasts predict a 70% surge in new cases and a 72% increase in global pancreatic cancer-related deaths by 2040. This review explores the intrinsic metabolic reprogramming of pancreatic cancer, focusing on the mevalonate pathway, including cholesterol biosynthesis, transportation, targeting strategies, and clinical studies. The mevalonate pathway, central to cellular metabolism, significantly shapes pancreatic cancer progression. Acetyl coenzyme A (Acetyl-CoA) serves a dual role in fatty acid and cholesterol biosynthesis, fueling acinar-to-ductal metaplasia (ADM) and pancreatic intraepithelial neoplasia (PanIN) development. Enzymes, including acetoacetyl-CoA thiolase, 3-hydroxy-3methylglutaryl-CoA (HMG-CoA) synthase, and HMG-CoA reductase, are key enzymes in pancreatic cancer. Inhibiting HMG-CoA reductase, e.g., by using statins, shows promise in delaying PanIN progression and impeding pancreatic cancer. Dysregulation of cholesterol modification, uptake, and transport significantly impacts tumor progression, with Sterol O-acyltransferase 1 (SOAT1) driving cholesterol ester (CE) accumulation and disrupted low-density lipoprotein receptor (LDLR) expression contributing to cancer recurrence. Apolipoprotein E (ApoE) expression in tumor stroma influences immune suppression. Clinical trials targeting cholesterol metabolism, including statins and SOAT1 inhibitors, exhibit potential anti-tumor effects, and combination therapies enhance efficacy. This review provides insights into cholesterol metabolism’s convergence with pancreatic cancer, shedding light on therapeutic avenues and ongoing clinical investigations.

## 1. Introduction

Pancreatic cancer represents currently 8% of cancer-related deaths and is the fourth leading cause in the United States [1]. The 5-year relative survival rate is still only around 11% [1]. According to the International Agency for Research on Cancer (IARC), the global number of annual new cases and pancreatic cancer-related deaths from 2020 to 2040 is predicted to increase by 70% (496,000–844,000) and 72% (466,000–801,000), respectively [2]. Deregulating cellular metabolism and immune evasion are some of the core hallmarks of cancer [3]. It is evident that cancer cells need to keep generating cellular components such as DNA, proteins, and lipids to enable rapid cell growth [4]. It has been demonstrated that the tumor-adjacent exocrine tissue exhibits upregulation of proteins related to lipid transport, which is associated with shorter post-operative survival in pancreatic cancer patients [5]. Cholesterol is an essential structural component of cell membranes and is important for physiological function [6]. 7-dehydroxycholesterol is the precursor for vitamin D and cholesterol, which itself is the key precursor for several important molecules such as bile acids as well as hormones such as glucocorticoid, mineralocorticoid, progesterone, estrogen, and testosterone [7]. Yet, growing evidence indicates that increased cholesterol flux is a common feature of cancer, and targeting the cholesterol biosynthesis pathway has been considered a promising therapeutic strategy [8]. In this review, we discuss metabolic reprogramming in pancreatic cancer as well as during pancreatic cancer development, focusing on the mevalonate pathway, cholesterol biosynthesis, transport, targeting strategies, and clinical studies.

## 2. The Role of the Mevalonate Pathway and De Novo Cholesterol Synthesis in Pancreatic Cancer

Acetyl coenzyme A (Acetyl-CoA) is the central molecule that participates in fatty acid synthesis as well as cholesterol biosynthesis [4]. Acetyl-CoA abundance is elevated in acinar cells of the pancreatic cancer mouse model called KC (*Pdx1-Cre*; *lox-stop-lox-Kras^G12D/+^*), and acetyl-CoA in the cholesterol biosynthesis pathway supports acinar-to-ductal metaplasia (ADM) formation [9]. ADM is the precursor for pancreatic intraepithelial neoplasia (PanIN), which can further progress to invasive pancreatic cancer [10]. Acetyl-CoA is also a substrate for histone acetyltransferases (HATs). Histone acetylation can lead to changes in gene expression associated with ADM formation. The oncogenic *Kras^G12D^* mutation is sufficient to promote histone H4 and histone H3 lysine 27 acetylation in acinar cells [9]. Histone acetylation marks are “written” by HATs and “read” by bromodomains cooperatively regulating transcription [11]. Bromodomain and Extra-Terminal motif (BET) protein inhibitor JQ1 prevent interaction between BET protein, acetylated histone, and transcription factors [12] and ADM formation [9]. These data emphasize the roles of acetyl-CoA and epigenetic regulations in the early stage of pancreatic carcinogenesis. Yet, the role of acetyl-CoA as a central source in cholesterol biosynthesis is also of significant importance in pancreatic cancer development, as the inhibition of cholesterol biosynthesis-associated enzymes attenuates ADM formation [9]. In this chapter, we will summarize and discuss the critical roles of enzymes involved in the mevalonate pathway and de novo cholesterol synthesis in pancreatic cancer.

**Figure 1:**
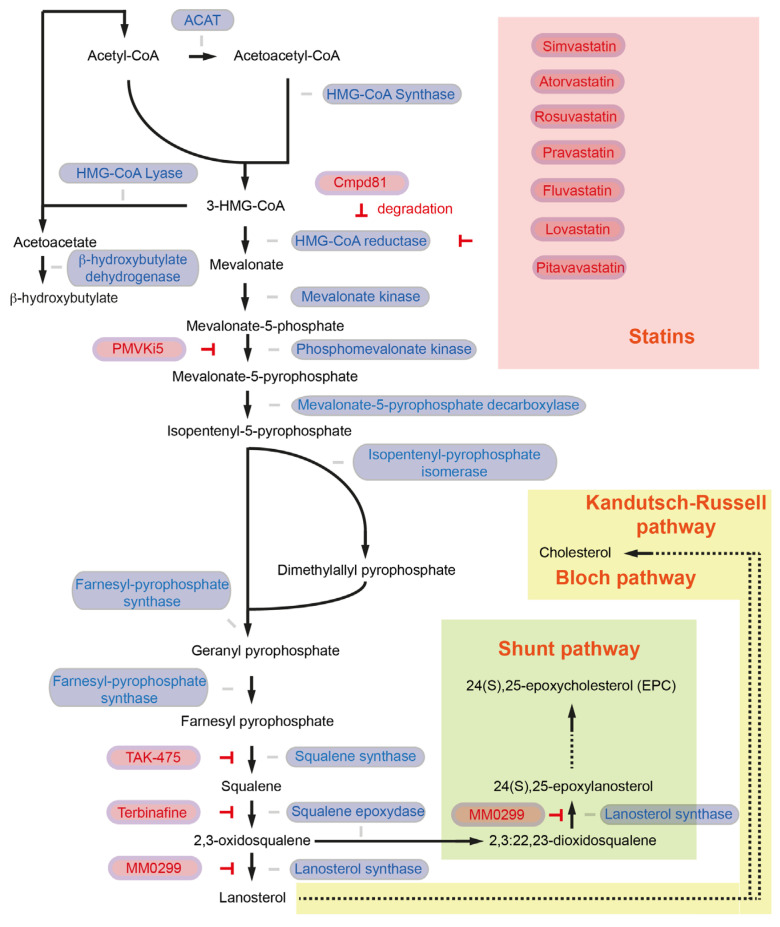
The cholesterol synthesis pathway. Enzymes involved in the reaction are colored in blue, and inhibitors and inhibition symbols are colored in red. Enzymes involved in ketone body biosynthesis, such as HMG-CoA lyase and β-hydroxybutylate dehydrogenase, are also included in the figure. ACAT: acetoacetyl-CoA thiolase; HMG-CoA: 3-hydroxy-3methylglutaryl-CoA.

In the first step, the enzyme acetoacetyl-CoA thiolase (also known as acetyl-CoA acetyltransferase, ACAT1 in mitochondria, ACAT2 in the cytosol) catalyzes a process to generate acetoacetyl-CoA (C4) from two acetyl-CoA molecules [6] (Figure 1). Sterol *O*-Acyltransferase 1 (encoded by the *SOAT1* gene, also known as Acyl-CoA cholesterol acyltransferase), which converts excess cholesterol to inert cholesterol esters, is also known as “ACAT1”. In the current review article, however, ACAT indicates acetyl-CoA acetyltransferase but not acyl-CoA cholesterol acyltransferase. ACAT1 is activated in several cancer types. Y407 phosphorylation of the ACAT1-tetramer by epidermis growth factor (EGF) stabilizes the active ACAT1-tetramer and supports cancer cell proliferation and tumor growth [13]. ACAT1 also possesses lysine acetyltransferase activity, which acetylates pyruvate dehydrogenase (PDHA1) and pyruvate dehydrogenase phosphatase (PDP1), leading to inhibition of the pyruvate dehydrogenase complex (PDC) activity [14]. As PDC plays a key link between glycolysis and the tricarboxylic acid (TCA) cycle, inhibition of PDC activity contributes to the Warburg effect [14]. The role of ACAT2 in pancreatic cancer and cancer development has not been fully elucidated. So far, it has been shown that elevated *ACAT2* gene expression is associated with radiotherapy resistance in pancreatic cancer cells [15]. Since the cytosolic ACAT2 enzyme is involved in cholesterol synthesis but the mitochondrial ACAT1 plays a more prominent role in cancer, it may be possible that cancer cells take advantage of ACAT2-mediated attenuated PDC activity rather than enhanced ACAT2 activity for cholesterol synthesis.

Subsequently, 3-hydroxy-3methylglutaryl-CoA (HMG-CoA) synthase (HMGCS1) catalyzes the condensation of acetoacetyl-CoA and acetyl-CoA molecules to form HMG-CoA (C6) and CoA [6] (Figure 1). Expression of the *HMGCS1* gene is higher in pancreatic cancer patients, which is associated with shorter disease-free survival [16]. CRISPR-Cas9-mediated knockout of *HMGCS1* suppresses the proliferation of gastric cancer cells [17]. Further, HMGCS1 can induce transcriptional upregulation of pluripotency genes *POU5F1* (*Oct4*) and *SOX2* [17] and is a key mediator of cancer stem cell enrichment in breast cancer [18]. These data suggest that HMGCS1 contributes to cancer in metabolic and non-metabolic ways. HMGCS1 drives drug resistance and is suggested to serve as a target for the treatment of acute myeloid leukemia patients [19]. Hymeglusin (L-659,699) is the specific HMGCS1 inhibitor [20], and hymeglusin enhances the therapeutic efficacy of venetoclax in acute myeloid leukemia [21]. Whether hymeglucin can inhibit pancreatic cancer development and progression needs to be clarified.

Conversion of HMG-CoA to acetoacetate and acetyl-CoA is catalyzed by HMG-CoA lyase (encoded by the *HMGCL* gene). HMGCL is a key enzyme in ketogenesis. Acetoacetate will be converted into β-hydroxybutylate catalyzed by β-hydroxybutylate dehydrogenase, which plays an important role in pancreatic cancer. HMGCL protein level is high in pancreatic cancer in mice (*Pdx1-Cre*; *lox-stop-lox-Kras^G12D/+^*; *Ink4a/Arf^lox/lox^*). HMGCL and β-hydroxybutylate contribute to pancreatic tumor aggressiveness and support metastatic dissemination [22]. Further studies to explore the molecular mechanisms regulated by HMGCL are required to validate HMGCL as a druggable candidate to target pancreatic cancer [22].

HMG-CoA reductase (*HMGCR*) regulates the conversion of HMG-CoA to mevalonate. HMGCR is the NADPH-dependent rate-limiting enzyme in the mevalonate pathway and is important for the further generation of the isoprenoid products (geranyl pyrophosphate and farnesyl pyrophosphate) [6] (Figure 1). Pancreatic expression of *Hmgcr* is upregulated in a murine spontaneous pancreatic cancer model (*Pdx1-Cre*; *lox-stop-lox-Kras^G12D/+^*; *Ink4a/Arf^lox/lox^*) [23], as well as in pancreatic cancer patients [24]. As HMGCR is the master regulator in the mevalonate pathway for cholesterol synthesis, several HMGCR inhibitors, such as statins, have been considered as cholesterol-lowering agents as well as anti-cancer drugs. An HMGCR inhibitor, simvastatin (Table 1), delays PanIN progression in KC mice (*Pdx1-Cre*; *lox-stop-lox-Kras^G12D/+^*) and attenuates pancreatic cancer development in KPC (*Pdx1-Cre*; *lox-stop-lox-Kras^G12D/+^*; *lox-stop-lox-Trp53^R172H/+^*) mice [25]. Another HMGCR inhibitor, atorvastatin, also inhibits cancer development and increases the survival of KPC mice [26]. In addition to simvastatin and atorvastatin, several statins have been approved by the FDA, namely rosuvastatin, pravastatin, fluvastatin, lovastatin, and pitavastatin [27]. High expression of *HMGCR* is not associated with shorter survival in pancreatic cancer patients [24]. Yet, an updated meta-analysis of 26 studies with more than 170,000 pancreatic cancer patients suggests a significant decrease in the risk of pancreatic cancer with statin use [28]. Mechanistically, it has been shown that atorvastatin inhibits Akt signaling via the P2X7 receptor in human pancreatic cancer cells, and expression of *Akt* and the *P2rx7* gene is also down-regulated in atorvastatin-fed *Ptf1a-Cre*; *lox-stop-lox-Kras^G12D/+^* mice [29,30]. Statins further decrease PD-L1 expression via JNK upregulation and TAZ downregulation [31]. In a pancreatic cancer xenograft mouse model, combination therapy with simvastatin and anti-PD-1 showed an enhanced anti-tumor effect compared to simvastatin or anti-PD-1 mono-therapeutic treatment [31].

Statin treatment can, however, induce compensatory increases in *HMGCR* also in pancreatic cancer [32]. It has been shown that the compensatory and counter mechanisms of the cells to maintain cholesterol levels induce an epithelial-to-mesenchymal transition (EMT)-like cell state and trap pancreatic cancer cells in a mesenchymal-like state. Several pancreatic cancer cell lines are capable of ERK activation upon statin treatment, leading to increased metastatic seeding ability through enhanced migration, extravasation, and survival. Since cancer cells are trapped in a mesenchymal-like state, they are unable to undergo a mesenchymal-to-epithelial transition (MET), and therefore statins inhibit the formation of metastatic colonies [32]. To eliminate the statin-induced accumulation of HMGCR, a sterol analog as a potent HMGCR degrader named compound 81 has been identified [33]. Further studies are needed to clarify whether inhibition of compensatory accumulation of HMGCR can further decrease pancreatic cancer risk.

Mevalonate will be further phosphorylated by mevalonate kinase (*MVK*) and subsequently by phosphomevalonate kinase (*PMVK*) to form mevalonate-5-phosphate and mevalonate-5-pyrophosphate (C6), respectively. Mevalonate-5-pyrophosphate decarboxylase (also known as mevalonate diphosphate decarboxylase, *MVD*) catalyzes the reaction from mevalonate-5-pyrophosphate to generate isopentenyl-5-pyrophosphate (C5). Isopentenyl-5-pyrophosphate will be converted to demethylallyl pyrophosphate by isopentenyl-diphosphate isomerase (IDI) [6] (Figure 1). There have been two IDI members identified: IDI1 and IDI2, IDI1 is found in most eukaryotes [34]. In humans, it has been shown that IDI2 is expressed only in skeletal muscle [35]. Farnesyl diphosphate synthase (coded by the *FDPS* gene) catalyzes a chain elongation from isopentenyl-5-pyrophosphate and demethylallyl pyrophosphate to produce geranyl pyrophosphate (C10). FDPS forms farnesyl pyrophosphate (C15) from geranyl pyrophosphate and an additional isopentenyl-5-pyrophosphate molecule [6] (Figure 1).

The potential role of MVK, PMVK, MVD, IDI, or FDPS in pancreatic cancer has not been fully addressed. In the case of liver cancer, it has been shown that the PMVK protein level is higher in tumors than in non-tumor areas. High PMVK expression is associated with shorter survival in liver cancer patients. Mechanistically, PMVK phosphorylates β-catenin and enhances its stability. Mevalonate-5-pyrophosphate also stabilizes β-catenin by inhibition of casein kinase 1 alpha 1 (CKIa, encoded by the *CSNK1A1* gene)-mediated S45 phosphorylation of β-catenin. This prevents proteolytic degradation of β-catenin [36]. Hepatic *Pmvk* knockout or intraperitoneal administration of small PMVK inhibitor named PMVKi5 (C_24_H_23_ClN_2_O_6_,N-[(E)-[3-[(4-chloro-3,5-dimethylphenoxy)methyl]-4-methoxyphenyl]methylideneamino]-3,4,5-trihydroxybenzamide) attenuates hepatocarcinogen diethylnitrosamine and carbon tetrachloride-induced hepatocellular carcinogenesis [36]. In the case of prostate cancer, it has been shown that FDPS is associated with PTEN loss and Akt activation [37]. EGF-induced cancer cell invasion requires Ras homolog (Rho) GTPases-mediated actin-cytoskeltal reorganization. For membrane attachment and biological activity, Rho GTPases require posttranslational modifications to provide a lipophilic anchor [38]. Rho can be geranylgeranylated, and Ras can be farnesylated [39]. Oncogenic *KRAS* mutations are observed in more than 90% of pancreatic cancer patients; among those, the *KRAS^G12D^* mutation is the most common and present in nearly 40% of pancreatic cancer patients [40]. A small molecule *KRAS^G12D^* inhibitor MRTX1133 has been generated and specificity and efficacy have been preclinically proven [41]. Yet, targeting KRAS farnesylation to globally modulate KRAS activity could also be a therapeutic option for pancreatic cancer. KRAS requires farnesylation for membrane localization, and therefore farnesyltransferase inhibitors have been developed to block the membrane translocation. However, farnesyltransferase inhibitor treatment causes KRAS geranylgeranylation to become active; therefore, a dual farnesyltransferase and geranylgeranyl-transferase inhibitor named FGTI-2734 has been developed [42]. FGTI-2734 attenuates Akt, mTOR, and c-Myc signaling activity and inhibits the growth of pancreatic cancer patient-derived xenografts with *KRAS^G12D^* or *KRAS^G12V^* mutation [42]. Further preclinical and clinical studies are needed to validate the anti-cancer effects of FGTI-2734. The role of MVK, PMVK, MVD, IDI, or FDPS in producing geranyl pyrophosphate and farnesyl pyrophosphate in pancreatic cancer also needs to be clarified. Geranyl pyrophosphate and farnesyl pyrophosphate are key intermediates in cholesterol synthesis but also for post-translational modifications. Squalene synthase (also known as farnesyl-diphosphate farnesyltransferase 1, coded by the *FDFT1* gene) catalyzes a reductive dimerization of two farnesyl pyrophosphate molecules to form squalene (C30) in a NADPH-dependent manner [6] (Figure 1). Pancreatic cancer patients with high levels of *FDFT1* expression show significantly shorter overall survival [43]. Interestingly, in murine pancreatic cancer autochthonous model (*Ptf1a-Cre*; *lox-stop-lox-Kras^G12D/+^*; *Trp53^lox/+^*), CRISPR-Cas9 screening for targeting ca. 3000 metabolic genes identified *Fdft1* as one of the most differentially dependent genes in vivo. *Fdft1* knockout shows no decrement in 2D tumor cell proliferation but exhibits growth inhibition in 3D culture and orthotopic transplanted tumor cells [43]. Loss of *Fdft1* does not affect the famesylation of Ras but attenuates activation of the Akt signaling pathway [43]. Administration with an FDFT1 inhibitor TAK-475 (1-[2-[(3R,5S)-1-[3-(Acetyloxy)-2,2-dimethylpropyl]-7-chloro-5-(2,3-dimethoxyphenyl)-1,2,3,5-tetrahydro-2-oxo-4,1-benzoxazepin-3-yl]acetyl]-4-piperidineacetic acid, lapaquistat acetate) reduces subcutaneous transplanted pancreatic tumor cell growth and incubation of 3D pancreatic tumor cells with TAK-475 reduces activation of the Akt signaling pathway [43]. For colon cancer, it has been shown that patients with high FDFT1 protein expression show significantly shorter overall and relapse-free survival than patients with low FDFT1 expression [44]. 

Squalene epoxidase (also known as squalene monooxigenase, coded by the *SQLE* gene) NADPH-dependently oxidizes squalene to 2,3-oxidosqualese (squalene epoxide) [6] (Figure 1). Pancreatic cancer patients exhibit high expression of *SQLE*, and high expression of *SQLE* is associated with shorter overall survival and disease-free survival of pancreatic cancer patients, high protein expression of SQLE exhibits shorter overall survival than patients with low SQLE expression [45,46,47]. SQLE promotes the proliferation and invasion of pancreatic cancer cells [46]. Mechanistically, SQLE attenuates the unfolded protein response pathway and activates the Akt signaling pathway [47]. An antifungal drug terbinafine has been widely used as a SQLE inhibitor. Terbinafine attenuates SQLE-induced pancreatic cancer cell proliferation and invasion [46]. Further, terbinafine augments the sensitivity of several chemotherapeutic drugs (e.g., cisplatin, 5-FU, gemcitabine) in pancreatic cancer cells [48]. Transcription of *SQLE* is directly repressed by p53 [49]. For other tumor entities such as liver cancer, it has been shown that terbinafine attenuates high-fat diet-mediated liver cancer development in p53 knockout mice (*Alb-Cre*; *Trp53^lox/lox^*) [49]. Terbinafine reduces tumor incidence and tumor number in diethynitrosoamine-injected, high-fat high-cholesterol diet-fed *Sqle* transgenic mice (*Alb-Cre*; *Rosa26-lox-stop-lox-Sqle-IRES*) [50]. Colorectal cancer patients with high SQLE expression (both RNA and protein levels) also exhibit shorter overall survival than patients with low SQLE expression [51]. Terbinafine attenuates the cell viability of colorectal cancer organoids as well as xenotransplanted tumor development and enhances chemosensitivity to 5-FU or oxaliplatin treatment [51]. On the contrary, another study showed that the median survival of colorectal cancer patients with high *SQLE* mRNA levels is longer than those with low *SQLE* expression [52]. Taken together, the role of SQLE in cancer is cancer type-specific or context-specific. In the case of pancreatic cancer, tumor-promoting and chemotherapeutic drug resistance have been shown. 

Lanosterol synthase (encoded by the *LSS* gene) converts squalene epoxide to lanosterol [6] (Figure 1). In the final step, the conversion from lanosterol into cholesterol is catalyzed by a number of enzymes via the so-called Bloch pathway and the Kandutsch-Russell pathway, where, in several steps, NADPH is required [53]. MM0299 inhibits LSS and diverts sterol flux from the Bloch pathway into the Shunt pathway, leading to commutation in 24(S),25-epoxycholesterol (EPC) [54] (Figure 1). So far, whether MM0299 has anti-tumor effects in pancreatic cancer has not been clarified.

Taken together, enzymes involved in the mevalonate pathway and cholesterol synthesis contribute to ADM and PanIN formation as well as pancreatic cancer progression, metastasis, and chemoresistance. Some enzymes are additionally involved in the Warburg effect, post-translational and epigenetic regulations, and further investigation is needed to better understand the shared and distinct molecular function between each enzyme involved in the mevalonate pathway and cholesterol synthesis.

**Table 1:** Inhibitors related to cholesterol metabolism.

Intervention/Treatment	Target	Reference
Simvastatin	HMGCR	[25,27,55]
Atorvastatin	HMGCR	[26,27]
Rosuvastatin	HMGCR	[27]
Pravastatin	HMGCR	[27]
Fluvastatin	HMGCR	[27]
Lovastatin	HMGCR	[27]
Pitavastatin	HMGCR	[27]
Cmpd81	HMGCR (degradation)	[33]
PMVKi5	PMVK	[36]
TAK-475	FDFT1	[43]
terbinafine	SQLE	[46,48,50,51]
MM0299	LSS	[54]
Avasimibe	SOAT1	[56,57]
Surface anchor-engineered T cells with liposomal avasibime	SOAT1	[58]
CP-113,818	SOAT1	[57]
K-604	SOAT1	[57]
Nilotinib	SOAT1	[59]
Alirocumab	PCSK9	[60]
Evolocumab	PCSK9	[60]
R-IMPP	PCSK9	[55]
PF-06446846	PCSK9	[55]

## 3. Cholesterol Modification, Lipoproteins, Uptake, and Transport in Pancreatic Cancer

**Figure 2:**
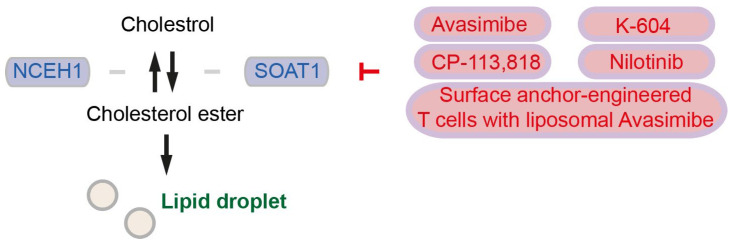
Enzyme involved in cholesterol and cholesterol ester conversion, as well as an inhibitor for SOAT1. Enzymes are colored in blue, and inhibitors and inhibition symbols are colored in red. NCEH: Neutral cholesterol ester, SOAT: Sterol *O*-acyltransferase.

Sterol *O*-acyltransferase 1 (SOAT1) converts excess cholesterol to inert cholesterol esters (CEs), which will be stored in lipid droplets (LDs) [56] (Figure 2). As we stated in Section 2, although SOAT1 is also known as acyl-CoA cholesterol acyltranferase 1 (ACAT1), in the current review, we term “SOAT” for sterol *O*-acyltransferase/acyl-CoA cholesterol acyltranferase and “ACAT” for acetyl-CoA acetyltransferase. LDs are dynamic cytoplasmic organelles and can be identified nearly ubiquitously in cells [61]. LDs highly accumulate in pancreatic cancer tissues, and LDs in pancreatic cancer cells contain high levels of CEs such as cholesteryl oleate and cholesteryl linoleate [56]. High CE accumulation and high *Soat1* expression are observed also in metastatic pancreatic organoids from KPC mice [62]. Patients with SOAT1 expression in pancreatic cancer (evaluated by immunohistochemistry) survived significantly shorter than patients without SOAT1 expression [56]. Cholesterol plays a role as the negative regulator for SREBP2 and the mevalonate pathway, but SOAT1-mediated conversion of cholesterol into CEs abrogates the cholesterol feedback mechanism that promotes mevalonate pathway dependency in pancreatic cancer [62]. CE accumulation is driven by PTEN loss and subsequent activation of the PI3K/Akt/mTOR signaling and activation of SREBPs [56]. Expression of SOAT1 is enhanced by p53 deficiency or even more by mutant p53, although mutant p53 is not necessary for up-regulation of SOAT1 [62]. Inhibition of cholesterol esterification by treatment with the SOAT1 inhibitor avasimibe reduces pancreatic cancer cell proliferation in vitro and in vivo, mechanistically suggesting that avasimibe enhances ER stress and apoptosis in pancreatic cancer cells [56]. Treatment with avasimibe or genetic ablation of *SOAT1* (mentioned as “*ACAT1*” in the publication) in T cells leads to potentiated effector function and enhanced proliferation in CD8^+^ T cells. In an orthotopic melanoma mouse model, avasimibe treatment attenuates tumor development leads to longer survival of mice, and further enhances the therapeutic efficacy of anti-PD-1 immunotherapy [57]. Surface anchor-engineered T cells containing liposomal avasimibe exhibited anti-tumor efficacy and enhanced survival in orthotopic melanoma and glioblastoma mouse models [58]. It needs to be clarified whether avasimibe surface anchor-engineered T cells show therapeutic effects in pancreatic cancer. In addition to avasimibe, CP-113,818, and K-604 have been identified to inhibit SOAT1 [57]. Further, ABT-737, evacetrapib, and nilotinib have been identified to bind SOAT1 proteins. SOAT1-targeting compounds increase the CD8^+^ T cell ratio to total immune cells, and nilotinib inhibits tumor activity in vitro and in vivo [59]. 

CEs can be catalyzed back to cholesterol by neutral cholesterol ester 1 (coded by the *NCEH1* gene), supporting cholesterol relocation from LDs to membranes [5] (Figure 2). It has been shown that high expression of *NCEH1* is associated with shorter overall survival of pancreatic cancer patients [45,63]. Increased NCEH1 protein abundances in the tumor-adjacent tissue, rather than in the neoplastic area, is associated with shorter survival of pancreatic cancer patients [5]. These studies suggest that both SOAT1 and NCEH1 play key roles in pancreatic cancer by regulating cholesterol availability. 

Triglycerides and to a lesser extent CEs are major components of very low-density lipoprotein (VLDL). Apolipoprotein C (ApoC), ApoE, and especially ApoB-100 are surface proteins of VLDL [64]. After dietary fat intake, lipoprotein particles called chylomicrons are generated and secreted by the intestine. Lipoprotein lipase (LPL) hydrolyzes triglycerides and transforms chylomicron into chylomicron remnants containing ApoB-48 and ApoE [65]. Chylomicron remnants will be taken up by the liver and metabolized. In hepatocytes, CEs and triglycerides are transferred to ApoB-100 in the endoplasmic reticulum where VLDL is produced [64]. Like LDs, VLDL arises close approximate to the endoplasmic reticulum and contains phospholipid membrane, triglycerides, and CEs. However, LDs contain different proteins such as LD-associated proteins [45]. LDs can be fused and enlarged, and LDs interact with other organelles such as endoplasmic reticulum, endosome, mitochondria, lysosome, and peroxisomes [61,66]. It has been suggested that lipoproteins may have evolved and gained secreting function from LDs, yet it is largely unknown how cells determine de novo synthesized lipids for storage in LDs or secretion [61], VLDLR recognizes ApoE-containing lipoproteins and is widely expressed but not in the liver [67]. LPL can also hydrolyze VLDL into intermediate-density lipoprotein (IDL, VLDL remnant) containing ApoB-100 and ApoE [65]. Triglycerides and phospholipids in IDL will be further hydrolyzed by hepatic lipase for generating low-density lipoprotein (LDL) containing ApoB-100 [65]. The LDL receptor (LDLR) recognizes ApoB-100 and ApoE thereby mediating the uptake of chylomicron remnants, IDL, and LDL [65]. 

LDLR has a physiologically important function. Mutations in the *LDLR* gene and impaired LDLR function lead to familial hypercholesterolemia, extremely elevated serum LDL levels, and the early onset of atherosclerosis [67]. Lipids can function as antigens (lipid antigens) and regulate the immune system [68]. The uptake of LDL-lipid antigen complexes by antigen-presenting cells is mediated by LDLR. Induced natural killer T cells driven by *LDLR*-mutant peripheral blood mononuclear cells from patients with familial hypercholesterolemia show impaired activation and proliferation upon ligand stimulation [69], highlighting the physiological importance of the LDLR. Yet, it has been shown that *Ldlr*, *Apob*, and *Apoe* genes are upregulated in pancreatic cancer in an oncogenic mouse model (*Pdx1-Cre*; *lox-stop-lox-Kras^G12D/+^*; *Ink4a/Arf^lox/lox^*) compared to control mice [23]. High *LDLR* expression is observed in all stages of pancreatic cancer and is associated with an increased risk of pancreatic cancer recurrence [23]. *LDLR* knockdown by shRNA prevents activation of ERK signaling in primary pancreatic cancer cells isolated from the mouse model and increases the sensitivity of pancreatic cancer cells to gemcitabine [23]. These data suggest that pancreatic cancer cells reprogram cholesterol metabolism and increase the uptake of LDL by upregulating *LDLR* expression. Further, pre-clinical studies are needed to clarify whether global LDLR inhibition is more beneficial—attenuating pancreatic cancer without significant side effects—or targeting LDLR specifically in pancreatic cancer is necessary. To that end, a cyclic molecule called VH4127 that specifically binds to the EGF homology domain of LDLR has been developed, and this peptide is further conjugated with a polyethylene glycol spacer, Myc-tagged Fc fusion protein, and the Alexa Fluor 680 dye for in vivo imaging purposes (named Fc(A680)-VH4127) [70]. Interestingly, Fc(A680)-VH4127 can be detected in pancreatic cancer and liver metastasis, but not in non-tumor pancreas or hepatocytes [70], potentially enabling tumor-specific drug delivery in the future.

**Figure 3:**
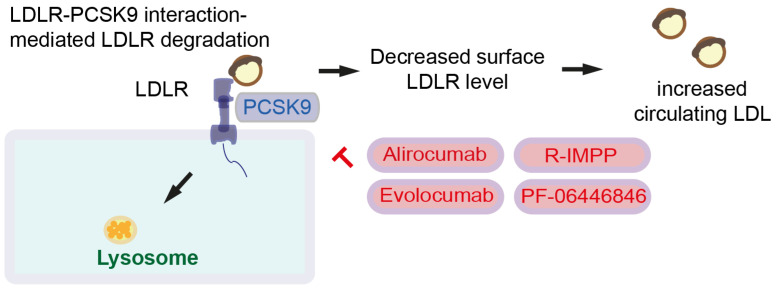
PCSK9 inhibitors. PCSK9 forms complex with LDLR and LDL for lysosomal degradation. This results in decreased levels of surface LDLR expression leading to increased level of circulating LDL. PCSK9 inhibitors and inhibition symbols are colored in red. LDL: Low-density lipoprotein, PCSK9: Proprotein convertase subtilin/kexin type 9.

LDLR is degraded after binding to a secretory serine protease named proprotein convertase subtilin/kexin type 9 (PCSK9). Together with LDL and LDLR, PCSK9 forms a complex and induces lysosomal degradation. Reduced surface expression of LDLR leads to increased circulating LDL. Hence, PCSK9 increases circulating LDL [60] (Figure 3). PCSK9 also binds CD36 to promote platelet activation and thrombosis [60]. Since high *LDLR* expression is observed in pancreatic cancer [23], high PSCK9 expression and LDLR degradation might be beneficial for pancreatic cancer patients. However, it has been shown that high PCSK9 protein levels are associated with shorter survival in colorectal cancer mice with *Apc* and *Kras* mutations (*Villin-Cre*; *Kras^G12D/+^*; *Apc^Min/+^*), but not mice with only the *Apc* mutation (wildtype *Kras*). In line with this, overexpression of *PCSK9* predicts shorter survival for colorectal cancer patients with *APC* and *KRAS* mutations, but not for colorectal cancer patients with only the *APC* mutation [55]. PCSK9 in colon cancer cells regulates EMT and PI3K signaling, enhancing tumor progression and lung metastasis [71]. Taken together, PCSK9-mediated global LDLR degradation is not beneficial for cancer patients; rather, tumor-specific LDLR targeting drug delivery is important. Several PCSK9 inhibitors, including monoclonal antibody inhibitors, such as alirocumab and evolocumab, have been generated [60]. Evolocumab and small molecule inhibitors (R)-N-(isoquinolin-1-yl)-3-(4-methoxyphenyl)-N-(piperidin-3-yl)propanamide (R-IMPP) as well as N-(3-Chloropyridin-2-yl)-N-((3R)-piperidin-3-yl)-4-(3H-[1,2,3]triazolo [4,5-b]pyridin-3-yl)benzamide (PF-06446846) inhibits CRC growth. Further, R-IMPP and simvastatin synergistically inhibit colorectal cancer xenograft growth [55].

High expression of *APOE* is associated with shorter survival in pancreatic cancer patients [72]. High *APOE* expression is observed in myeloid cells, especially in tumor-associated macrophages (TAMs) and fibroblasts. Subcutaneously injected melanoma and glioblastoma cells exhibit accelerated tumor growth and elevated levels of circulating and intratumoral myeloid-derived suppressor cells in global *Apoe* knockout mice [73]. On the contrary, it has also been shown that *APOE* ablation (global knockout mice) increases CD8^+^ T cells and reduces tumor burden after implantation of KPC cells into the pancreas. APOE promotes immune suppression in pancreatic cancer [72]. These contradictory findings suggest that whether APOE plays a tumor-promoting or -suppressing role may be tumor-type-dependent and context-dependent. The main evidence for some inhibitors in cancer is summarized in Table 2.

## 4. Targeting Cholesterol Metabolism and Clinical Trials

Targeting cholesterol metabolism, particularly the mevalonate pathway, has emerged as a promising therapeutic strategy for pancreatic cancer. As we discussed in the previous sections, statins, commonly used as cholesterol-lowering drugs, have shown efficacy in inhibiting pancreatic cancer growth and delaying cancer progression in animal models. Furthermore, statins have been found to inhibit signaling pathways and sensitize pancreatic cancer cells to other anticancer treatments. Combination therapies of statins and bisphosphonates have also demonstrated improved outcomes [74]. Clinical trials are currently investigating the effectiveness of various drugs, including statins (atorvastatin, simvastatin), ezetimibe, and other combinations (chemotherapy agents as FOLFIRINOX, simvastatin with metformin and digoxin), for the treatment of pancreatic cancer (Table 3). It is crucial to consider adverse events and safety concerns when using statins or other drugs, as statins may promote the basal type of pancreatic cancer associated with a poor prognosis [75]. Personalized treatment approaches that consider specific tumor characteristics and molecular profiling may be necessary to optimize therapeutic strategies and minimize potential side effects. Beyond pancreatic cancer, cholesterol metabolism has implications for other types of cancer. Studies have demonstrated that statins and other inhibitors of cholesterol metabolism can influence cancer progression and may have potential therapeutic applications in various malignancies [76]. The effects of statins on cancer cells extend beyond cholesterol metabolism and involve oxidative stress, DNA repair, isoprenylation of Ras proteins, and modulation of gene expression [76,77,78].

## 5. Conclusions

In conclusion, the mevalonate pathway and its components play crucial roles in the development and progression of pancreatic cancer. Targeting cholesterol metabolism, particularly through the use of statins, holds promise as a therapeutic strategy. Ongoing clinical trials are exploring the efficacy of various molecules and drug combinations for the treatment of pancreatic cancer. However, personalized treatment approaches and careful consideration of adverse events are necessary to optimize treatment outcomes. Further research is needed to unravel the complex molecular mechanisms and interconnections within cholesterol metabolism and explore its therapeutic implications not only in pancreatic cancer but also in other malignancies. A comprehensive understanding of the complex molecular mechanisms and therapeutic strategies associated with cholesterol metabolism is essential for the development of effective treatments for pancreatic cancer. Further investigation is required to explore the intricate interconnections between different components of the pathway and their roles in the development and progression of pancreatic cancer, paving the way for novel therapeutic interventions and improved treatment outcomes.

## Figures and Tables

**Table 2:** Role of inhibitors related to cholesterol metabolism in cancer.

Cholesterol Metabolic Gene	Role in Cancer	Inhibitor	Reference
*HMGCR*	Upregulated expression in pancreatic cancer mouse model and patients; HMGCR inhibitors attenuate pancreatic cancer development	Simvastatin, Atorvastatin, Rosuvastatin, Pravastatin, Fluvastatin, Lovastatin, Pitavavastatin	[23,24,25,26,27,55]
*SOAT1*	Patients with SOAT1 expression in pancreatic cancer have significantly shorter overall survival; Inhibition of SOAT1 reduces pancreatic cancer cell proliferation	Avasimibe, K-604, CP-113,818, Nilotinib, Surface anchor-engineered T cells with liposomal Avasimibe	[56,58]
*LDLR*	High LDLR expression observed in all stages of pancreatic cancer; Associated with increased risk of pancreatic cancer recurrence	-	[23]
*PCSK9*	High PCSK9 protein levels associated with shorter survival of colorectal cancer mice; PCSK9 inhibitors inhibit CRC growth	Alirocumab, R-IMPP, Evolocumab, PF-06446846	[55,60]
*APOE*	High APOE expression associated with shorter survival of pancreatic cancer patients	-	[72]

**Table 3:** Clinical trials regarding the use of drugs targeting the mevalonate pathway for pancreatic cancer patients.

Intervention/ Treatment	Condition or Disease	NCT Number	Stage of Clinical Trial	Recruitment Status (Recruiting, Completed, not Yet Recruiting. Last Update)	Last Update
Atorvastatin Evolocumab Ezetimibe FOLFILINOX	Metastatic pancreatic cancer	NCT04862260	Early Phase 1	Recruiting	7 June 2023
Simvastatin Metformin Digoxin	Advanced pancreatic cancer	NCT03889795	Phase 1	Recruiting	17 November 2021
Simvastatin Gemcitabine	Pancreatic cancer	NCT00944463	Phase 2	Completed	17 February 2017
Valproic acid Simvastatin Gemcitabine Nab paclitaxel Cisplatin Capecitabine	Untreated Metastatic Pancreatic Adenocarcinoma	NCT05821556	Phase 2	Recruiting	15 June 2023
